# The formation of a functional retinal pigment epithelium occurs on porous polytetrafluoroethylene substrates independently of the surface chemistry

**DOI:** 10.1007/s10856-017-5926-3

**Published:** 2017-07-13

**Authors:** Victoria R. Kearns, Jack Tasker, Zhuola, Riaz Akhtar, Akash Bachhuka, Krasimir Vasilev, Carl M. Sheridan, Rachel L. Williams

**Affiliations:** 1 Department of Eye and Vision Science, Institute of Ageing and Chronic Disease, University of Liverpool, Liverpool, UK; 2 Department of Mechanical, Materials and Aerospace Engineering, School of Engineering, University of Liverpool, Liverpool, UK; 3 School of Engineering, University of South Australia, Mawson Lakes, Adelaide, SA 5095 Australia

## Abstract

**Abstract:**

Subretinal transplantation of functioning retinal pigment epithelial (RPE) cells may have the potential to preserve or restore vision in patients affected by blinding diseases such as age-related macular degeneration (AMD). One of the critical steps in achieving this is the ability to grow a functioning retinal pigment epithelium, which may need a substrate on which to grow and to aid transplantation. Tailoring the physical and chemical properties of the substrate should help the engineered tissue to function in the long term. The purpose of the study was to determine whether a functioning monolayer of RPE cells could be produced on expanded polytetrafluoroethylene substrates modified by either an ammonia plasma treatment or an *n*-Heptylamine coating, and whether the difference in surface chemistries altered the extracellular matrix the cells produced. Primary human RPE cells were able to form a functional, cobblestone monolayer on both substrates, but the formation of an extracellular matrix to exhibit a network structure took months, whereas on non-porous substrates with the same surface chemistry, a similar appearance was observed after a few weeks. This study suggests that the surface chemistry of these materials may not be the most critical factor in the development of growth of a functional monolayer of RPE cells as long as the cells can attach and proliferate on the surface. This has important implications in the design of strategies to optimise the clinical outcomes of subretinal transplant procedures.

**Graphical Abstract:**

## Introduction

The retinal pigment epithelium (RPE) is a monolayer of cells located between the underlying choriocapillaris and the overlying neurosensory retina and is critical for the survival and function of both these structures. Degenerative changes in the RPE monolayer and its underlying basement membrane (Bruch’s membrane) lead to Age-related macular degeneration (AMD). AMD is the leading cause of blindness in subjects older than 50 years of age in the developed world. There are two types of AMD: neovascular (wet) and non-neovascular (dry). Despite substantial progress in the development of new therapies for wet AMD, the severe visual impairment associated with geographic atrophy in dry AMD remains untreatable [1, 2]. Replacement of the diseased RPE cells with healthy transplanted RPE cells is a feasible approach for a new AMD therapy [3, 4].

Transplantation of a suspension of cells has been demonstrated to be an unsuitable approach resulting in disappointing outcomes because aged human Bruch’s membrane does not support attachment, survival and differentiation of transplanted RPE [5], causing serious complications such as proliferative vitreoretinopathy [6]. An approach to circumvent this problem is to transplant a RPE sheet intact from the outset on an underlying substrate that mimics Bruch’s membrane. A number of biostable synthetic membranes that satisfy the physical properties required of a suitable transplanting device are currently being advocated [4, 7]. The physical properties required include biostability, porosity and suitable mechanical strength for surgical handling. It is well known that the surface properties of the underlying substrate directly influences the cells’ ability to form a differentiated monolayer [8]. It is highly likely that the production of a stable basement membrane by RPE cells grown on a synthetic membrane will be crucial to the long-term behaviour of the transplanted construct. Extracellular matrix (ECM) deposition by the RPE is likely to be affected by numerous parameters ranging from the surrounding biological environment to the underlying surface chemistry and topography to which the cells are exposed.

Expanded polytetrafluoroethylene (ePTFE) is a substrate that has many of the required physical properties of a transplanting device. It has a similar architecture to Bruch’s membrane, however it cannot support cells without surface modification due to its hydrophobic surface chemistry. The use of plasma technologies presents the opportunity to maintain the porous, fibrous structure of ePTFE while varying surface chemistry. Our previous work has investigated the deposition of thin polymer coatings via plasma polymerisation and direct modification of surface chemistry via ammonia plasma treatment. Both of these methods can be used to modify polymer substrates in a way that can support RPE growth and proliferation [9, 10]. Here we have investigated the effect of these surface modifications on a commercially-available, ePTFE-based substrate. The aims of the study were to determine whether the functionality conferred by these two modification methods could support a differentiated monolayer of RPE cells, and whether the difference in surface chemistries resulted in any alteration in the functional behaviour of the cells and the ECM that they produce over time. We have demonstrated that, although the surface chemistry of the ePTFE resulting from these two processes is very different they both support a functional monolayer of primary human RPE cells and that the underlying basement membrane produced on both surfaces in the longer term is similar.

## Methods

### Substrates

Substrates were 12 mm diameter Millicell^®^ culture plate inserts (Millicell-CM, Millipore (UK) Ltd., Watford). These are ePTFE membranes subjected to a proprietary treatment by the manufacturer and were designated UT-ePTFE_M. Virgin ePTFE and PTFE sheets (Goodfellow Cambridge Ltd., Huntingdon, UK) were also used as control substrates in some studies.

### Ammonia plasma treatment

Some UT-ePTFE_M, ePTFE and PTFE substrates were subsequently ammonia plasma treated with an in-house built helical resonator plasma system. This system and its operation have been described previously [11] and the operating conditions have been optimised to defluorinate the surface while causing minimal surface etching [12]. Immediately after plasma treatment, substrates were immersed in de-ionised, uv-sterilised water for at least 12 h to introduce polar groups to the surface [13]. These substrates were designated “NH_3_-ePTFE_M”, “NH_3_-ePTFE” or “NH_3_-PTFE”. Substrates were air-dried prior to further use.

### n-Heptylamine coating

Some UT-ePTFE_M and PTFE substrates were coated with *n*-Heptylamine (HA). The coating procedure was performed as described previously [14]. HA deposition was carried out for 40 s with power of 40 W. The pressure during deposition was 0.2 Torr. These substrates were designated “HA-ePTFE_M” or “HA-PTFE”.

### SEM

Substrates were sputter coated with chromium using an Emitech K575x with a chromium target (125 mA for 4 min). These were then imaged using a LEO 1550 field emission SEM (Zeiss, Welwyn Garden City, UK) using the secondary electron or in-lens detector, an accelerating voltage of 5 keV or 10 keV and a working distance of approximately 8–10 mm. Manual measurements of fibre and node diameters were obtained by ImageJ [15].

### Atomic force microscopy

Substrates were mounted on to 15 mm circular glass cover slips, then attached to metal specimen support discs using adhesive for positioning in the atomic force microscope (AFM). They were imaged with a Bruker Multimode AFM (NanoScope VIII, Bruker Nano Inc., Nano Surfaces Division, Santa Barbara, CA) using a 150 × 150 × 5 μm scanner (J-scanner). All test were conducted with the Peakforce Quantitative Nanomechanical Mapping (PFQNM) method [16]. Bruker RTESPA-150 silicon probes, with a nominal spring constant of 5 N/m and a tip radius of 8 nm, were used. For the nanomechanical property testing, the deflection sensitivity, spring constant of the cantilever and the tip radius were calibrated. A photostress polymer with a known elastic modulus (PS1, Vishay Precision Group, Heilbronn, Germany) was used to calibrate the elastic modulus. At least five areas were scanned on each ePTFE substrate and a minimum of three technical replicate samples were tested. The size of each image was 10 × 10 μm with a resolution of 384 pixels/line. The scan rate was 0.606 Hz. Data were analysed using Bruker Nanoscope Analysis software v. 1.5.

### Contact angle

The contact angle measurement was conducted using the static sessile drop method. Contact angles were measured using a drop shape analysis system (DSA100, Krüss). Three microliter water droplets of degassed and deionised water were dropped onto the surface. Images of the droplet were recorded over 10 s at 25 frames per second and the contact angle was determined from the first image in which the droplet was complete using the circle method. Contact angle measurements were performed on three areas on dry substrates. Substrates were tested in triplicate.

### XPS

ePTFE substrates were analysed using a Scienta ESCA300. This employs a high power rotating anode and monochromatised Al Kα X-ray source (hν = 1486.7 eV), high transmission electron optics and a multichannel detector [17]. Samples were covered with a mask and oriented at 45° to the beam to reduce charging. Charge compensation, optimised for each sample, was also used. The x-ray source was operated at 14 kV, 100 mA (1.4 kW) for survey and region scans. Survey spectra were recorded at 150 eV pass energy and 1.9 mm slitwidth, whereas region spectra were recorded at 150 eV pass energy, 0.8 mm slitwidth.

### Primary cell culture

Primary ocular tissue was collected under the host department’s ethical approval for the programme “Matricellular and related proteins in anomalous ocular repair and related processes; a program of study; LREC 01/066. Primary human RPE cells (hRPE) were isolated and expanded as described previously [9] and seeded onto substrates at 8.3 × 10^4 ^cells cm^−2^. Control substrates were tissue culture plastic coverslip (Sarstedt Ltd., Leicester, UK). Cells were seeded in F10 medium (Sigma-Aldrich Ltd., Dorset, UK) containing 2 mM L-glutamine, 50 U/ml penicillin G, 50 μg/ml streptomycin, 2.5 ug/ml amphotericin B, and supplemented with 20% foetal bovine serum (FBS). At day 2, FBS was reduced to 5% and medium was supplemented with 5 μM all-trans retinoic acid (Sigma-Aldrich Ltd). Medium was changed thrice-weekly.

### Immunocytochemistry

Confirmation of the epithelial status of isolated RPE cells was demonstrated by staining cells using a pan-cytokeratin antibody (details of all antibodies and concentrations are found in Table 1). Only these cells were used in further experiments. For investigation of cell morphology and cell-cell junctions, samples were fixed with 10% neutral-buffered formalin at days 7, 14, 21 and 28. For pan-cytokeratin and ECM studies, samples were fixed in 100% ice-cold methanol. Samples were permeabilised with Triton X-100 if formalin-fixed. Samples were blocked in 10% normal goat serum for 30 min at 37 °C then incubated overnight at 4 °C with the relevant antibody diluted in a 1% BSA: PBS solution. Samples were subsequently incubated with the appropriate secondary antibody for 60 min at 37 °C. Some formalin-fixed samples were counterstained with Alexa Fluor^®^ 488 phalloidin (Life Technologies, Paisley, UK). All samples were mounted with Vectashield^®^ Mounting Medium with DAPI (Vector Laboratories UK Ltd., Peterborough, UK). Samples were visualised using laser scanning confocal microscopy and associated Image Explorer software (LSM 500; Carl Zeiss).

**Table 1:** Details and dilutions of antibodies used in this study

Antigen	Antibody details; supplier	Dilution
Pan-CK, Clone C-11	Cat. # C9231; Sigma	1:200
ZO-1	Cat. # 40–2200; Invitrogen	1:100
Occludin	Cat. # 71–1500; Invitrogen	1:100
N-cadherin	Cat. # ab18203; Abcam (Cambridge, UK)	1:100
Fibronectin	Cat. # F0916; Sigma	1:100
Collagen I	Cat. # ab34710; Abcam	1:250
Collagen IV	Cat. # C1926; Sigma	1:100
Laminin-111	Cat. # L9393; Sigma	1:100
Alexa Fluor^®^ secondary antibodies	Invitrogen; various	1:500

### Dextran transport assays

Fluorescently-conjugated dextran solutions were made up in serum-free F10-HAM medium containing all other supplements. 10 kDa (D1976, Invitrogen), 70 kDa (FD70, Sigma-Aldrich) and 155 kDa (T1287, Sigma-Aldrich) dextrans were used to test a range of molecule sizes. Primary human RPE were seeded onto substrates as described above and grown for 28 d. Medium was removed and cell culture inserts were moved to new 24-well plates. Four hundred microlitres of dextran solution at a concentration of 50 μg/mL was added to the inner chambers of the inserts. Six hundred microlitres of serum-free medium was added to outer chambers. Plates were incubated at 37 °C. At 4, 8, and 24 h, 50 μL solution from outer chamber was removed and placed in 96-well black plates. Fifty microliter fresh medium was added to outer chambers. Plates were read at the appropriate wavelength for the fluorescent conjugate. Data were corrected against a medium blank. Samples were tested in triplicate.

### Statistical methods

Statistical analyses of the data were conducted in SPSS v.21 (IBM Corp., Armonk, NY). For AFM data elastic modulus data, a one-way ANOVA, followed by Tukey’s HSD post-hoc test, was conducted. For contact angle studies, a one-way ANOVA, followed by Tamhane’s T2 post-hoc test. For dextran transport assays a one-way ANOVA followed by Dunnett’s T3 post-hoc test was conducted.

## Results

### SEM

**Fig. 1:**
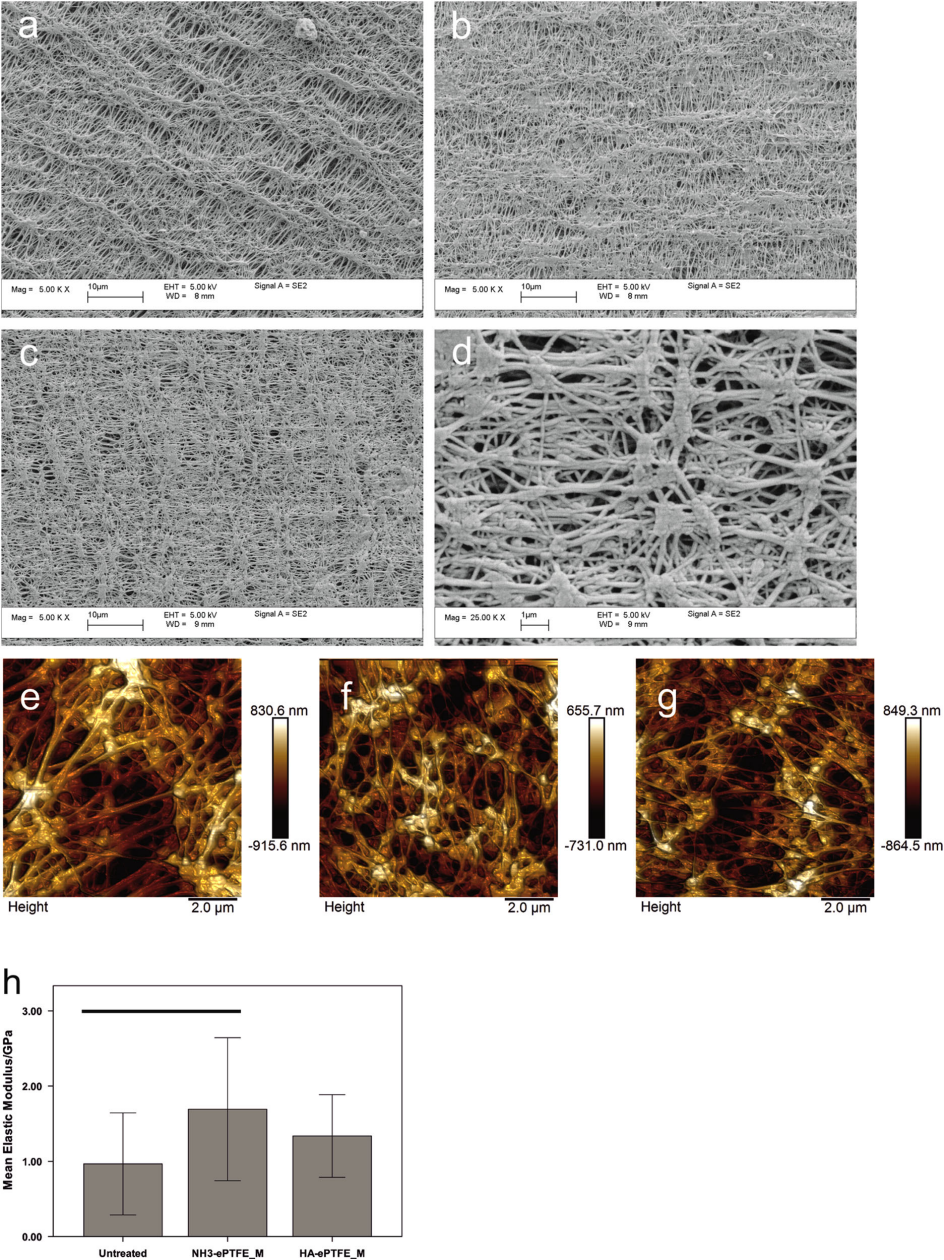
SEM micrographs of **a** UT-ePTFE_M, **b** NH_3_-ePTFE_M, and **c**, **d** HA-ePTFE_M show the node and fibre structure of the substrates. Surface treatment did not appear to have altered the macrostructure of the substrates. Atomic force microscopy images of **e** UT-ePTFE_M, **f** NH_3_-ePTFE_M, and **g** HA-ePTFE_M show the node and fibre structure of the substrates. Surface treatment did not appear to have altered the macrostructure of the substrates. The mean elastic modulus (**h)** increased following surface modification, but was only statistically significant (*P* ≤ 0.05) for NH_3_-ePTFE_M. Statistically significant differences are indicated by horizontal lines, error bars ± 1 standard deviation

SEM micrographs demonstrated that substrates had a fibrous structure, with fibres being connected by nodes and with fibres being aligned in some regions (Fig. 1a). Nodes measured between 1 and 2 μm. Fibre diameter was in the range 100–300 nm. NH_3_-ePTFE_M (Fig. 1b) and HA-ePTFE_M (Fig. 1c) did not appear to have a different structure, indicating that the two surface treatments had not caused surface etching or gross occlusion of the pores (representative image, HA-ePTFE_M, Fig. 1d).

### AFM

AFM images showed that UT-ePTFE_M, NH_3_-ePTFE_M and HA-ePTFE_M all had a similar fibre and node structure (Fig. 1e–g), supporting the assertion that surface modification had not resulted in alteration of the macrostructure or pore occlusion. Both ammonia plasma treatment and HA-coating increased the mean elastic modulus compared with the untreated substrates (Fig. 1h), although this difference was only statistically significant (*P* = 0.033) for NH_3_-ePTFE_M.

### Contact angle

Non-porous untreated PTFE substrates had the highest water contact of 95.0 ± 4.2°. Both ammonia plasma treatment and HA-coating reduced the contact angle (to 68.4 ± 5.4°, *P* ≤ 0.001 and 83.0 ± 3.9°, *P* ≤ 0.001), respectively, with the ammonia plasma treatment having the greatest effect.

For porous substrates, untreated ePTFE had the highest water contact angle (133.1 ± 4.7°), with that of UT-ePTFE_M being significantly lower (75.8 ± 4.2°, *P* ≤ 0.001). UT-ePTFE_M samples also allowed the water to penetrate the surface of the membrane. Ammonia plasma treatment of ePTFE_M (i.e. NH_3_-ePTFE_M) maintained this water penetration and lead to a significant reduction in contact angle (68.5 ± 4.0°, *P* = 0.023), whereas heptylamine deposition lead to a significant increase in contact angle (123.5 ± 0.8°, *P* ≤ 0.001). Untreated ePTFE and HA-ePTFE_M did not allow the water droplet to penetrate the surface. These surfaces also had contact angles significantly higher than their non-porous equivalents (*P* ≤ 0.001 in both cases) whereas there was no significant difference between porous and non-porous ammonia treated surfaces (*P* = 1.0).

### XPS

**Fig. 2:**
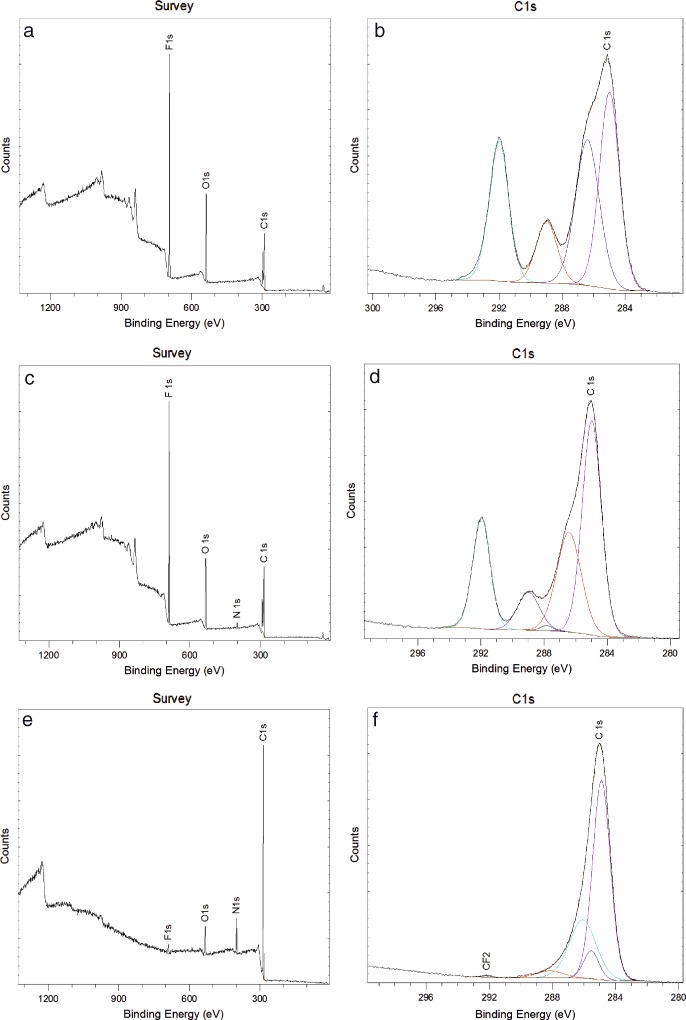
XPS survey and C1s region spectra for UT-ePTFE_M (**a**, **b**), NH_3_-ePTFE_M (**c**, **d**), and HA-ePTFE_M (**e**–**f**). The relatively small contribution from CF2 on the UT-ePTFE_M **b** indicates a prior surface treatment. Ammonia plasma treatment lead to the introduction of a small N1s peak (**c**). The n-heptylamine coating masked the underlying substrate properties, as demonstrated by the almost complete absence of fluorine signals (**e** and **f**)

The survey spectrum of UT-ePTFE_M (Fig. 2a) demonstrated the presence of a relatively large (contributing to 23% of the elemental peaks) O1s peak in addition to the F1s and C1s peaks (52 and 25% respectively), confirming that the material had been subjected to a modification treatment by the manufacturer. Relative atomic concentrations for regions identified on survey spectra are shown in Table 2 and those for the C1s region spectra in Table 3. The high resolution C1s spectrum (Fig. 2b) gave additional information on this surface. In addition to the CF_2_ peak at 292.0 eV (approximately 24% of surface species) and broad C1s envelope around 285 eV, there was a distinct peak around 289 eV, contributing about 11% of surface species. The C1s envelope comprised two separate peaks at 285.0 eV (34 % of surface species) and 286.4 eV (30% of surface species). The second of these peaks is attributed to oxygenated hydrocarbon (C-O) species.

**Table 2:** Relative atomic concentration in regions identified from survey spectra. UT-ePTFE_M exhibited a relatively large O1s peak, suggesting that this was not untreated ePTFE. NH_3_-ePTFE_M was similar, but with the addition of a small nitrogen peak. HA-ePTFE_M had a large reduction in the F1s contribution, a moderation reduction in the O1s peak and increases in C1s and N1s regions

	Concentration (atomic %)			
	C1s	N1s	O1s	F1s
UT-ePTFE_M	25.36	–	23.11	51.53
NH_3_-ePTFE_M	26.4	1.75	21.87	49.98
HA-ePTFE_M	71.71	11.29	12.03	4.97

**Table 3:** Contributions to C1s region spectra. The relatively low CF_2_ contribution in UT-ePTFE_M indicated a prior surface treatment. The spectra for UT-ePTFE_M and NH_3_-ePTFE_M were similar. A distinct peak around 289 eV was assigned to the C–F bond may be a result of the surface treatments breaking some of the C–F bonds and the introduction of oxygen functionality or from the bulk. HA-ePTFE peak assignment suggested that the signal from the bulk had been masked. A larger aliphatic carbon (C–C/C–H) contribution is thought to be from the alkyl chain in the surface coating

	Contribution (%)				
Peak	284.89	286.4	287.6	288.99	292.11
Assigned species	C–C/C–H	C–O/C–N	C=O	289	CF_2_
UT-ePTFE_M	34.14	30.6	–	11.24	24.02
NH_3_-ePTFE_M	43.69	25.99	0.66	9.00	20.66
HA_ePTFe_M	68.08	26.27	5.31	–	0.34

The survey spectrum for NH_3_-ePTFE_M (Fig. 2c) was similar to that of ePTFE_M in terms of peaks and their relative contributions, but with the addition of a small nitrogen peak (1.8%), which was expected [13]. The high resolution C1s spectrum (Fig. 2d) also exhibited similar peaks to ePTFE_M, with the C–C contributing around 43.7% of surface species, peaks at 286.4 eV (attributed to various C–O and C–N moieties, 26%) and 287.8 eV (attributed to C=O, 0.6%) and the CF_2_ peak at 292.0 eV (approximately 24% of surface species). The peak around 289 eV was again identified, contributing 9%.

The HA-ePTFE_M survey spectrum (Fig. 2e) was notably different to the UT-ePTFE_M and NH_3_-ePTFE_M. The F1s region contributed only around 5% of surface species (compared with around 50% for the other surfaces), and the C1s region increased to around 72%. The contribution from N1s region increased to approximately 11% and the O1s region contribution decreased to approximately 12%. In the C1s region scan (Fig. 2f) the C–C/C–H peak, at 284.9 eV, contributed 69.1%. Peaks attributed to amine, ether and other C–O and C–N moieties at 286.0 eV, C=O at 287.6 eV and CF_2_ at 292.1 eV were exhibited, contributing 26.3, 5.31 and 0.3% to surface species respectively. In contrast to the UT-ePTFE_M and NH_3_-ePTFE_M C1s spectra, no peak at 289 eV was identified.

### Cell morphology

**Fig. 3:**
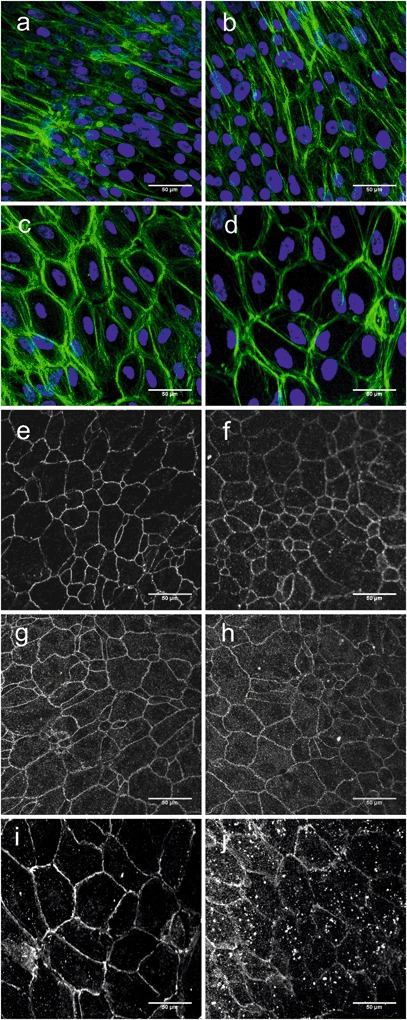
Photomicrographs of hRPE grown on NH_3_-ePTFE_M (**a**, **c**, **e**, **g**, **i**), and HA-ePTFE_M (**b**, **d**, **f**, **h**, **j**). At 7 days (**a**, **b**), cells on both substrates adopted an elongated morphology (cells were stained for F-actin, *green*, and counterstained with DAPI, *blue*) and appeared to conform to the underlying substrate topography. On both substrates at 28 days a cobblestone morphology was observed (**c**, **d**) and the formation of tight (**e**, **f**), occludens (**g**, **h**) and cadherins junctions (**i**, **j**) was confirmed with florescent immunostaining for ZO-1, occludin and n-cadherin. Scale bars represent 50 μm (color figure online)

At early time points, cells appeared to conform to the topography of the surface-modified ePTFE_M substrates, exhibiting an elongated morphology (demonstrated by visualisation of F-actin), even when confluent and irrespective of the surface treatment, (Fig. 3a, b). By 28 d, however, cells adopted an epithelial, “cobblestone” morphology (Fig. 3c, d) with some remaining stress fibres. Tight (Fig. 3e, f), occludins (Fig.3g, h) and adherens (Fig. 3i, j) junctions were observed. There appeared to be little qualitative difference between the two treated substrates in terms of cell morphology and cell-cell junction staining.

### ECM

**Fig. 4:**
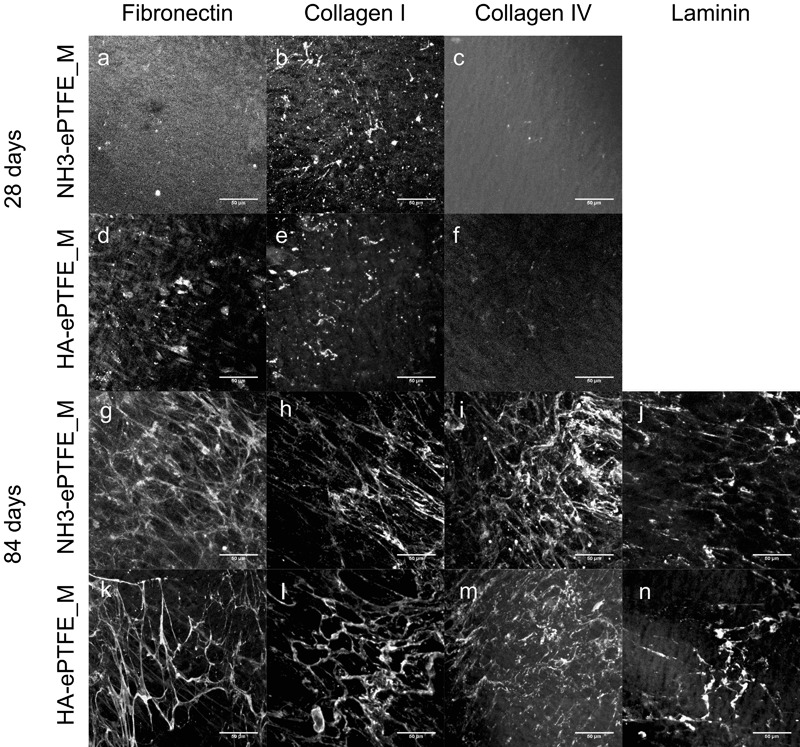
Photomicrographs of ECM expression on NH_3_-ePTFE_M (**a**–**c**, **g**–**j**) and HA-ePTFE_M (**d**–**f**, **k**–**n**). Samples were stained for fibronectin (**a**, **d**, **g**, **k**), collagen type I (**b**, **e**, **h**, **l**), collagen IV (**c**, **f**, **I**, **m**) and laminin-111 (**j**, **n**). A limited amount of ECM was observed at 28 d (**a**–**f**), and the surface topography can be seen in several images e.g. (**a**, **c**). No positive laminin staining was observed. Following 84 days in culture, both substrates demonstrated a fibril expression of fibronectin (**g**, **k**), collagen type I (**h**, **l**), and basement membrane components collagen IV (**i**, **m**) and laminin-111 (**j**, **n**). Scale bars represent 50 μm

For all of the proteins studied, little or no positive staining was observed at time points before 28 d. At 28 d, limited protein deposition was detected for fibronectin, collagen I and collagen IV, and, where present, it had a globular or limited fibrillar appearance (Fig. 4a–f). No positive staining for laminin alpha 1 was observed at 28 d. Culture periods were extended for up to 84 d. Some evidence of a fibronectin network was seen on both surfaces at 56 d. A well-formed network over the surface was found at 84 d (Fig. 4g, k). Similar behaviour was observed for collagen 1 (e.g. 84 d Fig. 4h, l). A collagen IV network was observed on HA-ePTFE_M surfaces at 56 d but not on NH_3_-ePTFE_M, although it was at 84 d on that surface (Fig. 4m). Laminin still had a patchy, globular appearance at 56 d, and a limited network formation even at 84 d (Fig. 4j, n).

**Fig. 5:**
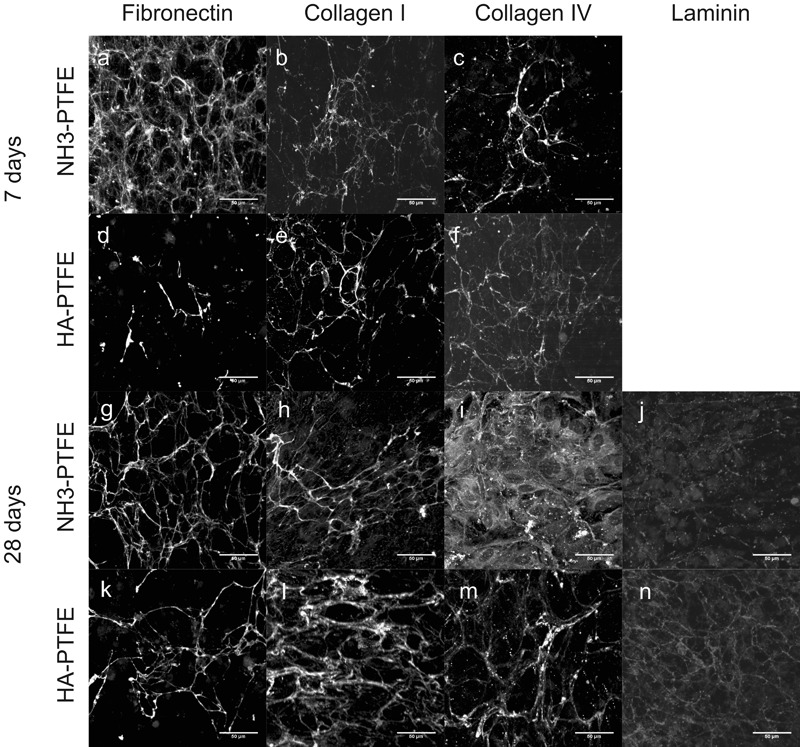
Photomicrographs of ECM expression on non-porous NH_3_-PTFE (**a**–**c**, **g**–**j**) and HA-PTFE (**d**–**f**, **k**–**n**). After 7 day culture of hRPE (**a**–**f**), primitive ECM networks were observed on both substrates. At 28 d (**g**–**n**), denser ECM networks were detected, with limited laminin deposition at this time point. Scale bars represent 50 μm

In order to separate the effect on protein deposition of culture on porous surfaces from the effect of the surface modifications, cells were grown on PTFE (i.e. non-porous) substrates (NH_3_-PTFE and HA-PTFE) subjected to the same surface treatments. In contrast to the behaviour observed on ePTFE_M, protein networks were observed much earlier. For fibronectin, small patches of fibrils were seen on HA-PTFE surfaces at 7 d (Fig. 5d), but on NH_3_-PTFE, a more fibrous network arrangement was seen (Fig. 5a). By 28 d, a network was observed on both surfaces (Fig. 5g, k), although appeared to be more well-formed on NH_3_-PTFE. For collagen types I and IV, patches of fibrillar protein were observed at 7 d on both surfaces (Fig. 5b, c, e, f), with a more comprehensive network found at 28 d (collagen 1 Fig. 5h, l; collagen IV Fig. 5i, m). Notably, a primitive laminin network was observed at particularly on HA-PTFE surfaces (Fig. 5j, n), which is in contrast to that seen on the equivalent ePTFE_M substrate.

### Dextran transport assays

**Fig. 6:**
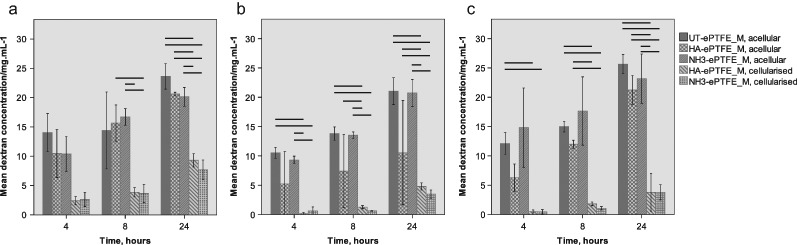
Concentration of 10 kDa (**a**), 70 kDa (**b**) and 155 kDa (**c**) dextran passing through ePTFE_M substrates. Error bars ± 1 standard deviation. Statistically significant (*P* ≤ 0.05) differences are indicated by horizontal lines. In all cases, the amount of dextran that passed through the filters increased with time. There was no significant difference between the amount of dextran that passed through substrates with cells on them

All sizes of dextran could be transported through the substrates, whether or not cells were present. Less dextran passed through substrates when cells were present than through their acellular equivalent. Statistical analysis confirmed this was the case for all sizes of dextran at 24 h (Fig. 6). At earlier time points the statistical significance of the results is less clear, but the trend suggests that the presence of cells reduces dextran transport. No difference was found between the amounts of dextran passing through acellular substrates at any time point. Similarly, there was no difference in the dextran permeability between the two cellular substrates. These data indicate that the surface treatment had no effect on dextran transport, either in the presence or absence of cells. It appeared that, in every case, the amount of dextran that passed through the substrates increased with time, indicating that pores were not being occluded; this was not tested statistically due to the small sample size.

## Discussion

Treating AMD by implantation of a functioning monolayer of RPE, or RPE-like cells, delivered on a carrier substrate, offers huge potential. In addition to resolving the issue of the best cell source for this application, understanding of the optimal substrate properties is required in order to support the cells pre- and post-implantation. The most important requirements of the substrate are that they support the attachment and growth of a monolayer of functional RPE cells and continue to support the cells post implantation in the long term. It is well known that the surface wettability, chemistry and topography will influence the attachment of cells to a substrate. Once the cells have attached they will begin to secrete ECM molecules which will become incorporated in to the basement membrane between the substrate and the cells. We hypothesise that to achieve the long term stability and functioning of the transplanted cells that the basement membrane should mimic the Bruch’s membrane of the native retinal pigment epithelium. The question that arises is whether the surface properties of the substrate influence the composition of the basement membrane produced in the long term and thus the stability of the RPE monolayer.

ePTFE has a similar architecture to Bruch’s membrane but cannot support cell attachment without surface modification [9]. The use of plasma technologies allows us to maintain the porous, fibrous structure of ePTFE while varying surface chemistry by plasma polymerisation or by direct modification of surface chemistry via ammonia plasma treatment. Qualitative examination of the surfaces using SEM and AFM suggested that neither the ammonia plasma treatment nor the addition of a heptylamine coating lead to changes in the surface topography of individual fibres or the porosity of the membrane. For the ammonia plasma treatment the conditions have previously been optimised and have been reported not to cause surface etching of PTFE [18]. In contrast to a previous report [19], deposition of a plasma polymer coating did not occlude the pores. It was important to characterise the as-received ePTFE membranes (UT-ePTFE_M). The measurement of a water contact angle was lower than that of untreated ePTFE and the ability of water to penetrate the surface and the presence of oxygen functionalities on the XPS indicated that this material had been subjected to a proprietary surface treatment and was not virgin ePTFE. The absence of nitrogen functionality suggests that this treatment was not ammonia or nitrogen gas plasma treatment [12]. O_2_ and Ar gas plasma treatment of PTFE is reported to result in the incorporation of oxygen functionalities without nitrogen functionalities [12, 20]; similar treatments may have been used to produce UT-ePTFE_M. Studies investigating the effect of the ammonia plasma treatment on PTFE have reported defluorination [11], evidenced by a large reduction in the F1s peak. In the current study, when ammonia plasma treatment was used on UT-ePTFE_M to produce NH_3_-ePTFE_M, the extent to which defluorination was observed was not as great as those previous reports. This reflects the reduction of fluorine which had already been caused due to the proprietary treatment.

For UT-ePTFE_M and NH_3_-ePTFE_M there was a distinct peak in the high resolution C1s spectra around 289 eV assigned to the C–F bond similar to a peak identified by Wilson et al. [13]. This is possibly due to the treatment breaking some, but not all, of the C–F bonds and the introduction of oxygen functionality or may be due to signal from the bulk. This peak was not identified on the HA-ePTFE_M and in conjunction with the large increase in C1s and N1s contributions, suggested that the HA coating masked the signal from the bulk ePTFE. Furthermore, analysis of the high resolution C1s region spectrum for HA-ePTFE_M indicated a larger aliphatic carbon (C–C/C–H) contribution, probably due to contributions from the alkyl chain in the surface coating. The peaks identified that are attributed to the HA coating are in agreement with those reported previously [21].

Contact angle analysis was used to give an indication of the effect of the different surface treatments on surface wettability. Contact angle experiments were conducted on non-porous substrates with the same surface treatments as their porous equivalents (with the exception of the proprietary treatment on the ePTFE_M), in order to determine the effect of surface chemistry on contact angle independently of the effects of the surface topography. Untreated virgin PTFE had the highest contact angle of the non-porous substrates, with NH_3_ plasma treatment leading to a significant reduction, as reported previously [13]. HA_PTFE_M were more hydrophobic than NH_3_-PTFE samples correlating with the hydrocarbon content measured by XPS, but not to the same extent as untreated PTFE. The values obtained here are in the range reported previously for flat n-heptylamine surfaces [22]. As expected, porous ePTFE substrates gave different values to their non-porous counterparts. The untreated ePTFE exhibited contact angles within the reported range [23, 24] and was more hydrophobic than the PTFE. Similarly, HA-ePTFE_M had a higher contact angle that HA-PTFE. These materials appear to behave according to the Cassie-Baxter model, with air being trapped in the pores, and the water droplet being pinned, resulting in a larger contact angle than that of the equivalent flat surface [25, 26]. This would also explain why the water droplet did not penetrate into the surface. NH_3_-PTFE and NH_3_-ePTFE_M surfaces had similar contact angles. This suggests that this material is not behaving according to either the Cassie-Baxter model or the Wenzel model, [25, 27] where the liquid would enter the pores and the droplet spread across the surface, resulting in a lower contact angle than for an equivalent non-porous substrate. One possible explanation is that the surface treatments do not modify the fibres inside the porous substrates to the same extent, so once water has entered the pores, it may not continue to infiltrate at the same rate, although we did observe that the droplet penetrated into the surface. Interestingly, complete wetting of all ePTFE_M substrates was possible, as demonstrated by the dextran transport studies, where liquid was applied to the upper and lower surfaces and molecules were able to penetrate the membranes.

Primary human RPE cells were able to form a confluent monolayer on both HA-ePTFE_M and NH_3_-ePTFE_M surfaces, despite their different surface wettabilities. This would suggest that the nitrogen and oxygen containing functional groups present in the HA-ePTFE_M surface were of sufficient concentration to promote cell attachment despite the overall hydrophobic nature of the surface. Cells on both surfaces adopted an epithelial phenotype, with the presence of cell-cell junctions and the ability to control the passage of dextran molecules through this monolayer demonstrating their functionality. Dextran molecules as large as 500 kDa can pass through Bruch’s membrane in vitro, although this decreases with age, particularly at the macula [28]. As expected, and as reported by others [29], the amount of dextran passing through the RPE-ePTFE_M constructs decreased as molecular weight increased. There was no difference in the amount of dextran passing through when the substrates were acellular, indicating that the difference is mediated by the cells and not the substrate. RPE cells are reported to be the dominant contributor to the barrier to molecules passing through the RPE-choroid complex [30]; our study demonstrated similar results, with the time taken for dextran molecules to pass through the RPE-ePTFE_M constructs being much longer than for the acellular substrates. No differences were observed between the behaviour on the HA-ePTFE_M and NH_3_-ePTFE_M surfaces. These data support our previous, preliminary, findings where several different surface treatments were able to support RPE proliferation [10]. Similarly, Sorkio et al. reported that a range of different ECM coatings on tissue culture plastic supported the formation of differentiated monolayers of embryonic stem cell-derived RPE [31]. This indicates that there may not be one optimal surface treatment, although other features such as epithelial maturity may be influenced by the surface chemistry [31]. Indeed, given that the community is still learning about the level of maturity required from implanted cells and the inherent heterogeneity of native RPE [32, 33], it may be difficult to identify a single ideal surface chemistry.

Surface architecture also appears to be important, and, where surface chemistry is sufficient to support appropriate cell attachment and growth, may be dominant over the effect of the surface chemistry. Studies have suggested that surface topography influences many aspects of cellular behaviour, including that of RPE cells [34]. These questions are not only relevant for the development of substrates for subretinal transplantation, but also for in vitro models. Epithelial cells are frequently cultured on substrates described as “transwells”, or “tissue culture inserts”, without description, or even consideration, of the surface chemistry and architecture in such devices. In the study by Liu et al. [35], the authors demonstrated that human foetal RPE were able to maintain characteristics of differentiated RPE better on two 200 nm fibrous substrates of different chemistries than smooth surfaces made from the same polymer, and suboptimal growth on 1000 nm diameter fibres, indicating that surface chemistry is not always the dominant factor, and that a similar response can be obtained on surfaces with different surface chemistries. The fibres in this study were of the same order of magnitude. On the other hand, in our study, cell morphology appeared to follow substrate topography when the cells were pre confluent, before adopting an epithelial, “cobblestone” morphology. This, coupled with the apparent absence of differences in cell behaviour on these surfaces, suggests that the influence of the surface architecture is not as significant once the cells have become confluent.

Surface mechanical properties are another important, yet frequently-overlooked mediator of cellular response [36]. The in vitro behaviour of RPE has been reported to be influenced by substrate stiffness [37], with the data suggesting that increasing stiffness leads to undesirable cellular responses. Studies of the mechanical properties of Bruch’s membrane are limited in number, study different layers and use a range of techniques to obtain data, however the elastic modulus appears to be around 2–4 MPa [38]. The substrates used in this study were several orders of magnitude higher than that of Bruch’s membrane, although similar to those used in other studies [7, 35] and in the same order of magnitude as that reported for non-porous PTFE [39]. Furthermore, the differences in surface stiffness resulting from the different surface treatments were relatively small, even across multiple regions on different samples. This may explain the similarity in cellular response that was observed. In future, it may be useful to develop substrates for RPE transplantation that have surface mechanical properties closer to those of Bruch’s membrane.

The formation of a stable basement membrane by RPE cells grown on a synthetic membrane is likely to be crucial to the long-term behaviour of the transplanted construct; extracellular matrix performs a range of roles and forms part of the cellular microenvironment. The apparent absence of ECM deposited on the surface of the porous substrates at early time points was a surprising finding. In contrast, ECM deposition on non-porous substrates with equivalent surface treatments appeared, qualitatively, to be similar to each other and even enhanced compared to that observed on control TCP substrates at these early time points. Even after several weeks in culture, ECM deposition on porous substrates was patchy and mostly disorganised in arrangement, compared to the networks observed on their non-porous counterparts. Only after many weeks did the distribution of ECM components on the porous substrates appear to be similar to that reported by Sorkio et al. [31] whereas on the non-porous substrates it was similar to that reported for growth on tissue culture plastic surfaces [40] from the early time points. The difference in cell behaviour on the porous and non-porous substrates in terms of the time taken to deposit ECM seems to be mediated by the porosity rather than surface chemistry in our case. We were able to use porous and non-porous surfaces subjected to identical surface treatments, although the porous ePTFE_M had been subjected to some proprietary pre-treatment. The size and flexibility of the ePTFE substrates made it difficult to quantify the deposited proteins, as standard methods require significant scraping of the surfaces to ensure the ECM components are removed [41]. It may be that at the early time points the deposited ECM becomes distributed within the surface pores and thus it takes longer for a structured basement membrane to become apparent. It is clearly important that the substrate is porous to allow transport of nutrients and waste across the RPE layer in vivo, however, these data may suggest that a non-fibrous porous membrane might be advantageous in terms of providing a surface for deposition of a structural basement membrane at an earlier time point. On the other hand, we demonstrated that a stable functional monolayer of primary human RPE cells was present on the porous treated membranes long before the deposited ECM had become organised suggesting that the organisation is not necessary at the early stage but that the cells continue to remodel their basement membrane with time.

## Conclusion

This study investigated two different surface modifications of an ePTFE-based substrate and found that they resulted in very different surface chemistry and wettability, while not appearing to modify macrostructure or topography. Both modifications supported the formation of a functioning monolayer of primary human RPE cells and the deposition of extracellular matrix components on each had a similar appearance. The time taken for the extracellular matrix to exhibit a network structure took months, whereas on non-porous substrates with the same surface chemistry, a similar appearance was observed after a few weeks. This study suggests that neither the specific surface chemistry, wettability or topography of these materials are critical to the growth of a functional monolayer of RPE cells as long as the cells can attach and proliferate on the surface initially. This conclusion fits with the literature which has demonstrated good in vitro growth of RPE and RPE-like cells on substrates with a range of very different surface properties. This has important implications on the design of strategies to optimise the clinical outcomes of subretinal transplant procedures.
